# Late Vertebral Metastasis in Long-Term Survivors of Breast, Prostate, and Renal Cell Cancer

**DOI:** 10.3390/cancers18213543

**Published:** 2026-11-04

**Authors:** John Berry-Candelario

**Affiliations:** Department of Neurosurgery-CU Anschutz School of Medicine and UCHealth-University of Colorado Hospital, Aurora, CO 80045, USA; john.berry-candelario@cuanschutz.edu

**Keywords:** vertebral metastasis, cancer survivorship, late recurrence, tumor dormancy, spinal cord compression, narrative review

## Abstract

Long-term survivors of hormone receptor-positive breast cancer, prostate adenocarcinoma, and renal cell carcinoma may develop metastatic disease years after treatment. Vertebral involvement can cause instability and neurologic injury, but first late vertebral recurrence is poorly measured because studies frequently combine vertebral and other skeletal events. This narrative review synthesizes selected recurrence cohorts, experimental studies, randomized trials, and professional guidance. It distinguishes first late distant recurrence, biochemical recurrence, and progression of established metastatic disease; examines proposed mechanisms of marrow persistence; and considers detection and management. Late systemic risk is established, particularly in estrogen receptor-positive breast cancer, but vertebral-specific incidence remains uncertain. Niche studies provide biological plausibility without demonstrating a uniquely vertebral dormancy process. MRI assesses local marrow and neural involvement, while tumor-appropriate PET/CT maps disease distribution; tracer biology influences detection. Relevant trials address different questions: spinal MRI screening in metastatic castration-resistant prostate cancer, treatment of identified high-risk bone lesions, or metastasis-directed therapy in oligorecurrent disease. None establishes scheduled spine MRI for asymptomatic disease-free survivors. Concerning symptoms and tumor-specific recurrence signals warrant timely diagnostic evaluation. Identified lesions require assessment of neurologic compression, oncologic options, mechanical stability, and systemic status. Prospective cohorts and intervention studies should measure vertebral-specific events, function, quality of life, and downstream harms before risk-defined screening is recommended.

## Simple Summary

Some cancers can return many years after treatment, including in the bones of the spine. These lesions may cause pain, fracture, or nerve injury. We review late recurrence after breast, prostate, and kidney cancer, explaining what long-term studies show and where spine-specific evidence is missing. We also examine how cancer cells may persist in bone and why those mechanisms do not yet justify routine spine scans for every survivor. New concerning back pain or neurologic symptoms require timely assessment, while a detected lesion needs coordinated evaluation by the cancer and spine teams. Better studies are needed to identify whether selected survivors benefit from earlier imaging without unnecessary tests or treatment.

## 1. Introduction

Improvements in cancer treatment have extended the period during which patients may live with a residual risk of metastatic recurrence. For survivors treated with curative intent, the passage of several disease-free years is reassuring but does not uniformly imply elimination of that risk. Estrogen receptor-positive breast cancer can recur well beyond the initial course of endocrine therapy, and renal cell carcinoma (RCC) can reappear after prolonged intervals following nephrectomy [1,2,3]. Prostate cancer presents a different survivorship pathway because serial prostate-specific antigen (PSA) testing may reveal recurrence before metastatic lesions become clinically apparent [4]. These trajectories make delayed vertebral involvement a relevant concern across oncology, primary care, rehabilitation, and spine practice.

The clinical importance of vertebral metastasis depends on anatomy as well as tumor burden. A lesion confined to marrow may be asymptomatic, whereas growth into the epidural compartment can threaten neural function. Destruction of load-bearing structures may produce mechanical pain, collapse, or deformity even without substantial epidural extension. Conversely, extensive skeletal disease does not necessarily imply instability at every involved level. Neurologic compression, local mechanical failure, and systemic progression are related but distinct problems. Their separation explains why a vertebral event can alter a survivor’s function and treatment needs disproportionately to its apparent contribution to total metastatic volume [5].

The epidemiology of delayed vertebral recurrence remains difficult to describe. Most long-term oncology datasets report distant recurrence or bone metastasis without identifying the vertebral level, epidural involvement, presentation, or structural consequences. Studies of patients referred for spinal surgery begin at a later point in the disease course and are enriched for symptomatic or complicated lesions. Neither type of dataset directly estimates the probability that an asymptomatic survivor will experience a first vertebral metastasis after a specified disease-free interval. A narrative review must therefore distinguish evidence for persistent systemic risk from evidence specifically describing vertebral events.

This review examines late recurrence in hormone receptor-positive breast cancer, prostate adenocarcinoma, and RCC because these malignancies illustrate different forms of delayed skeletal involvement and different follow-up approaches. We integrate clinical recurrence data with experimental evidence of marrow persistence, evaluate the arguments for spine-directed detection, and discuss management after a lesion is suspected or identified. The objective is to clarify actionable clinical vigilance and research priorities without converting incomplete epidemiologic or biological evidence into an unvalidated screening recommendation.

## 2. Review Approach and Definitions

This narrative review synthesizes clinical cohorts, randomized trials, mechanistic investigations, and professional guidance. The initial targeted search of PubMed and primary journal and guideline websites extended through 9 October 2026; focused searches addressing molecular imaging were updated through 28 October 2026. Search concepts combined late recurrence, vertebral or spinal metastasis, bone metastasis, tumor dormancy, breast cancer, prostate cancer, renal cell carcinoma, spinal cord compression, surveillance, and metastasis-directed treatment. The update added PET/CT, FDG, sodium fluoride, PSMA, MRI, and bone scintigraphy. Citation chaining identified foundational studies and relevant procedure guidelines. The literature assessed was in English; non-English publications were not systematically searched or translated. No lower publication-date limit was imposed for foundational evidence.

Selection prioritized studies with an explicit population, disease state, time origin, and clinically interpretable endpoint. Vertebral-specific cohorts, spinal imaging studies, and trials reporting compression, instability, or skeletal complications were prioritized for spine-specific claims. When direct evidence was unavailable, broader recurrence or skeletal-metastasis studies were retained as indirect evidence, and their anatomic limitations were identified. Comparative diagnostic studies and professional imaging guidance informed modality selection; randomized trials informed treatment or screening effects. Mechanistic studies were used to explain biological hypotheses, and isolated case reports were not used to estimate incidence or screening benefit. Imaging studies were interpreted in their original staging or recurrence populations rather than as studies of disease-free survivors. Selection was a qualitative judgment of relevance and applicability, without duplicate independent screening, a prespecified protocol, formal risk-of-bias assessment, or quantitative pooling.

We use late first distant recurrence to mean a newly recognized distant metastatic event more than five years after curative-intent treatment in a patient without previously diagnosed distant disease. Recurrence beyond ten years is described as very late. These thresholds are operational definitions rather than biological boundaries; individual studies may measure latency from diagnosis, surgery, treatment completion, or a five-year disease-free landmark. Their time origins must be preserved when interpreting results. A first vertebral event may occur simultaneously with other distant lesions and should not automatically be termed an isolated spinal recurrence.

Biochemical recurrence is a marker-defined state and is not synonymous with metastatic disease. Oligorecurrence describes a limited number of detected metastatic lesions after treatment of the primary tumor; oligoprogression describes limited progression against a background of established metastatic disease, commonly during systemic therapy. A patient developing a vertebral lesion ten years after prostatectomy may belong to the first category, while a patient whose known vertebral metastasis progresses on treatment belongs to the second. Combining these populations obscures both recurrence incidence and the purpose of imaging. Similarly, a vertebral lesion after a long remission requires consideration of a new primary malignancy or nonmalignant process rather than automatic attribution to the historical cancer.

## 3. Epidemiology and the Problem of the Denominator

Stating that a proportion of all cancer patients develop spinal metastases describes a different risk from the conditional probability of late recurrence among survivors who remain disease-free. The latter depends on selection at the landmark, subsequent competing mortality, duration of observation, and the original stage and biology. The proportion of recurrent cases occurring after ten years is also different from the proportion of ten-year survivors who later recur. These denominators are particularly important in RCC, where long-term series may select patients who survived sufficiently long to enter the analysis [2,3].

Anatomic reporting introduces further uncertainty. Bone metastasis, axial skeletal metastasis, and vertebral metastasis are overlapping endpoints, but they are not interchangeable. The pelvis and ribs may be included within an axial category, and vertebral involvement may be detected only after another site initiates staging. A useful spine-specific dataset would record first detection, whether the vertebra was the only metastatic site, whether pain or a neurologic deficit prompted imaging, and whether instability or epidural disease was present. Without these fields, one cannot infer the frequency of a preventable spinal emergency from the frequency of skeletal relapse. Accordingly, breast and RCC recurrence estimates in this review are presented as evidence of persistent systemic or skeletal risk, not as estimates of first late vertebral metastasis.

Referral and detection patterns can influence the apparent timing of recurrence. Surgical cohorts omit many patients treated with radiation or systemic therapy alone and patients with asymptomatic lesions. Greater use of sensitive molecular imaging may move the recorded date of metastatic recurrence earlier without changing the underlying biological onset. Changes in adjuvant treatment can likewise modify risk, so historical cohorts may not represent contemporary survivors. Table 1 summarizes these distinctions across the three cancers; the absence of a vertebral-specific rate is an evidence gap rather than evidence that the event is rare or common.

## 4. Tumor-Specific Patterns of Late Recurrence

### 4.1. Hormone Receptor-Positive Breast Cancer

The most robust long-term recurrence evidence comes from estrogen receptor-positive breast cancer. Pan and colleagues examined patients scheduled to receive five years of endocrine therapy and demonstrated continuing distant-recurrence risk during years five through twenty, with risk varying by original tumor size, nodal status, and grade [1]. This establishes the importance of late systemic relapse and the relevance of initial disease characteristics long after treatment. It does not provide a corresponding estimate of first vertebral recurrence, nor does it show that vertebral imaging improves outcomes in otherwise asymptomatic survivors.

Population-based follow-up further supports the persistence of risk beyond a decade. Pedersen and colleagues studied women who had remained disease-free for ten years and documented recurrence through year thirty-two, with greater risk associated with larger tumors, nodal involvement, and estrogen receptor positivity [12]. This landmark approach complements the endocrine-treatment cohort analyzed by Pan and colleagues, but the recurrence endpoint does not isolate vertebral disease. Molecular characterization also reveals heterogeneity within estrogen receptor-positive cancer: Rueda and colleagues identified genomic subgroups with distinct late-relapse trajectories [13]. These findings support biological stratification in future survivorship studies; they do not yet validate a genomic threshold for dedicated spinal imaging.

Trials of extended endocrine treatment show that the survivorship trajectory can be modified by treatment duration. ATLAS compared continuation of tamoxifen to ten years with stopping at five years, while MA.17R examined extension of aromatase-inhibitor therapy [14,15]. These studies inform systemic risk reduction, not selection of a spinal screening interval. A review of vertebral recurrence should acknowledge endocrine exposure and treatment-era differences rather than treating all patients with hormone receptor-positive disease as one homogeneous long-term risk group. Adherence and competing treatment toxicities also affect the clinical context in which later symptoms are evaluated.

Bone is a familiar site of breast cancer metastasis, and experiments support persistent disseminated cells within marrow niches [6,7]. However, the presence of a plausible marrow reservoir cannot identify which survivor will develop an actionable vertebral lesion. A sclerotic, lytic, or mixed focus may be discovered during systemic staging, while pain may have an alternative explanation such as degeneration or an insufficiency fracture. The differential is especially important when treatment-related bone loss and age-related fragility coexist. Clinical evaluation should address both malignant and nonmalignant explanations, with imaging interpreted alongside prior studies and the pattern of symptoms.

Clinical evidence of marrow dissemination complements these experimental observations. Braun and colleagues pooled individual patient data from nine studies of early-stage breast cancer and found that bone marrow micrometastasis at diagnosis was associated with poorer prognosis [16]. The association supports the relevance of occult disseminated disease, but a positive marrow finding cannot establish continuous dormancy, identify the eventual metastatic site, or determine when a vertebral lesion will become clinically consequential. Its prognostic significance should therefore remain distinct from evidence supporting a screening intervention.

Follow-up after curative treatment emphasizes history, examination, and breast surveillance; routine systemic imaging in asymptomatic patients is not supported by the ASCO follow-up guidance [10]. Adjuvant bisphosphonate evidence concerns recurrence and survival in selected patient groups and should not be interpreted as proof that late vertebral disease has been eradicated or that subsequent screening is unnecessary [17]. For this review’s clinical purpose, the defensible message is that a remote breast cancer history remains relevant when persistent or progressive spinal symptoms develop. The magnitude of long-term systemic risk can inform judgment, but cannot substitute for a validated vertebral risk model.

### 4.2. Prostate Adenocarcinoma

Prostate cancer differs from breast cancer because PSA provides a repeatable biochemical signal after local treatment. Rising PSA can prompt evaluation years after prostatectomy or radiotherapy, but the clinical significance of recurrence depends on the treatment received, PSA kinetics, pathology, and whether a management change is possible. EAU guidance does not support routine imaging after treatment of localized disease when PSA is not rising, and the patient is asymptomatic [4]. Follow-up in patients with established metastatic disease follows a different logic, and evidence from that setting should not be generalized to disease-free survivors.

PSMA PET has improved localization of recurrent disease. A prospective study of gallium-68 PSMA-11 PET evaluated its accuracy in patients with biochemical recurrence and illustrates how molecular imaging can detect disease not apparent on conventional staging [18]. Localization of a vertebral focus nevertheless does not characterize the degree of epidural compression or prove mechanical instability. These questions may require MRI, CT, and clinical assessment. Equivocal uptake should be correlated with anatomic findings rather than treated as sufficient evidence for a destructive vertebral metastasis requiring local intervention.

Experimental work demonstrates that prostate cancer cells can compete for occupancy of the hematopoietic stem-cell niche in mouse marrow [8]. This supports a model of skeletal colonization and persistence, but it does not establish a clinical latency distribution for vertebral metastases. Osteoblastic appearance also should not be equated with intact structural strength. The mechanical consequences of a lesion are determined by its location, alignment, fracture status, and involved structures, not solely by whether imaging appears sclerotic. These considerations remain relevant even when biochemical markers suggest a slowly evolving systemic course.

The ORIOLE phase II trial evaluated stereotactic ablative radiation versus observation in selected men with recurrent hormone-sensitive oligometastatic disease and found improved short-term disease-control outcomes with treatment [19]. Its relevance is to management after metastases have been identified, not to routine MRI screening or prevention of spinal emergencies. Oligorecurrence and oligoprogression also imply different systemic treatment decisions. A vertebral lesion arising during biochemical-recurrence staging may be one manifestation of a limited detected burden; a growing lesion during therapy for castration-resistant cancer represents another state. Table 2 separates these evidence populations to prevent a treatment benefit from being misrepresented as a screening benefit.

### 4.3. Renal Cell Carcinoma

RCC can recur after long disease-free intervals following nephrectomy. Kim and colleagues evaluated recurrence beyond five years in patients with localized disease, while the Japanese Society of Renal Cancer described events among patients followed beyond ten years [2,3]. Both provide evidence of prolonged risk, but neither establishes that the vertebra is the most frequent initial site of late recurrence. In the Japanese cohort, late events occurred in several organs, with bone reported as one category. Descriptions of extremely delayed lesions are useful demonstrations of possibility, but case reports cannot estimate their probability among all survivors.

A more recent retrospective cohort of 2960 patients with stage 1 or 2 RCC reported 211 recurrences, including 67 after five years and 14 after ten years; common first-recurrence sites included lung, bone, and the contralateral kidney [25]. These observations reinforce the need to retain late recurrence in the differential after nephrectomy. However, the median follow-up was 59 months, observation duration varied, and bone events were not resolved into vertebral-specific outcomes. The study consequently strengthens evidence for prolonged systemic risk without defining the conditional incidence of a first late spinal event.

Initial stage, grade, histology, and treatment context influence recurrence risk. Clear-cell RCC biology should not be assumed to apply to every RCC subtype, and a review centered on very late disease should avoid attaching one numerical risk to all nephrectomy patients. Survivors also accumulate competing health risks, including renal impairment, that affect the value and feasibility of imaging. EAU follow-up guidance uses recurrence-risk profiles and recommends individualization rather than uniformly abandoning imaging or prescribing lifelong high-intensity surveillance [11]. Thus, it would be inaccurate to state that follow-up across all three cancers is simply symptom-driven.

Once identified, an RCC vertebral metastasis may require attention to lytic destruction and potential vascularity. These features affect assessment of fracture risk, procedural bleeding, radiation planning, and surgical preparation. They do not imply that every RCC lesion is unstable or that preoperative embolization is required in every case. Decisions depend on anatomy, the proposed intervention, patient condition, and local expertise, and should be made within a multidisciplinary assessment [5]. A long interval since nephrectomy may make the presentation unexpected, but it does not replace systemic restaging or tissue confirmation when the diagnosis is uncertain.

The von Hippel–Lindau and hypoxia-inducible factor pathways are central to clear-cell RCC biology [9]. Their role in angiogenesis provides a plausible context for persistence and subsequent tumor growth. However, an established pathway in RCC development is not itself evidence that hypoxic vertebral marrow maintains a specific dormant reservoir for decades. Clinical claims about delayed vertebral reactivation should therefore remain separate from established molecular features of the primary tumor. The practical implication is recognition of possible late recurrence and appropriate evaluation, not a biologically inferred schedule of spinal imaging.

## 5. Dissemination and Persistence in the Marrow Environment

### 5.1. Vertebral Anatomy and Routes of Spread

The vertebral column contains vascular marrow and lies close to neural structures whose function can be compromised by a relatively small increment in local tumor growth. Batson’s work established the relevance of the vertebral venous system as one route of dissemination [26]. Venous anatomy offers a useful explanation of susceptibility, but it should not be presented as the exclusive or proven dominant pathway for every malignancy. Arterial dissemination and interactions between tumor cells and the marrow environment also contribute. Anatomic access, successful colonization, persistence, and later growth are separate steps in the metastatic process.

The consequences of vertebral growth depend on the occupied compartment. Marrow involvement may precede cortical destruction, and epidural extension may develop from an osseous lesion. Structural damage can cause pain with loading, while epidural disease can affect neural tissue before dramatic vertebral collapse occurs. The clinical endpoint of interest is therefore not merely the presence of a tumor cell or imaging focus. It is a lesion with a trajectory, location, and treatment opportunity that could alter patient outcomes. This distinction connects anatomical susceptibility with the uncertainty surrounding population screening.

### 5.2. Dormancy Is a Model Rather than a Retrospective Diagnosis

Dormancy may describe a nonproliferating disseminated cell, a small population with balanced proliferation and cell loss, or clinically undetectable disease held in check by its environment. These states cannot usually be distinguished in a patient presenting with a late vertebral lesion. A prolonged disease-free interval is compatible with dormancy, but may also reflect slow growth below imaging thresholds, treatment suppression, or a changing host environment. It is therefore more precise to describe dormancy as a plausible contributor to late recurrence than to state that late vertebral disease proves reactivation of a previously quiescent marrow cell.

**Figure 1:**
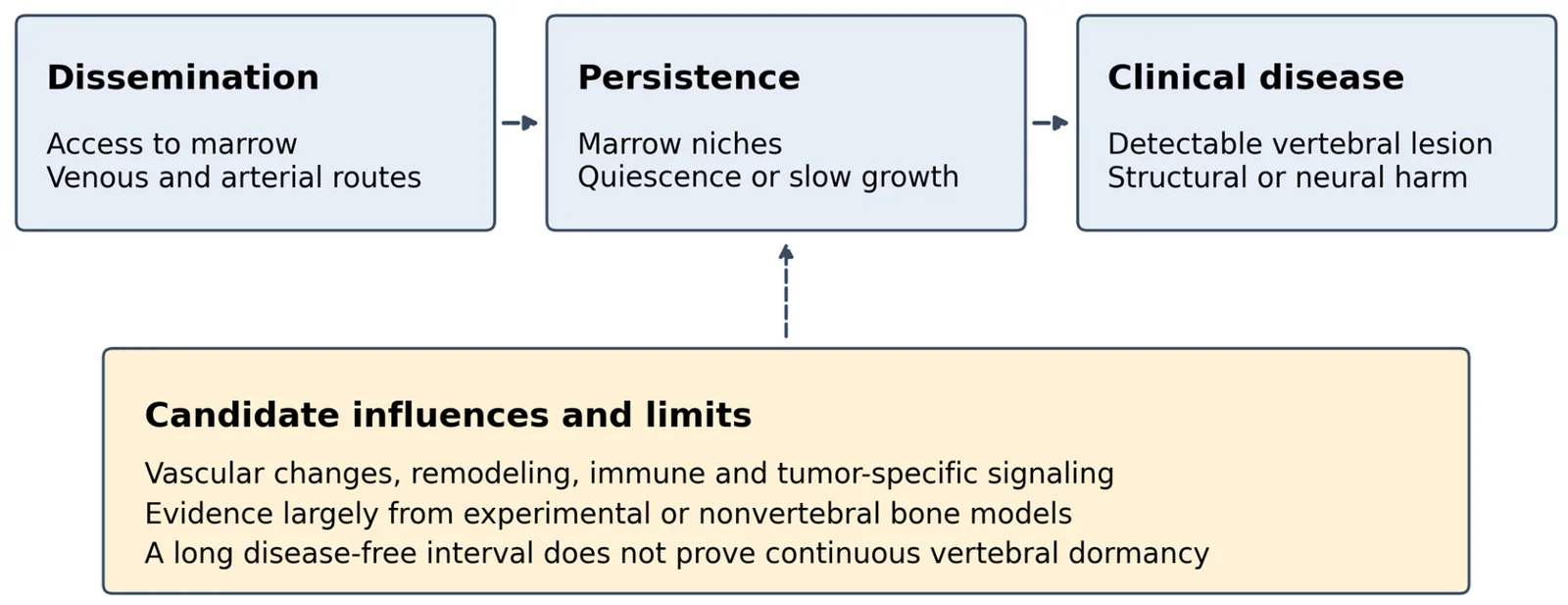
**Marrow persistence and late clinical disease.** Conceptual sequence from dissemination to marrow persistence and clinically detectable vertebral disease. Dashed arrows indicate uncertain transitions. Mechanistic evidence derives largely from experimental and nonvertebral marrow studies; prolonged remission does not prove uninterrupted dormancy at the vertebral site subsequently involved.

Preclinical breast cancer studies provide a foundation for this model. Ghajar and colleagues linked a stable perivascular environment with dormancy and vascular sprouting with growth-promoting conditions; Price and colleagues examined specific marrow niches involved in tumor-cell residence and transit [6,7]. These investigations identify mechanisms through which the local environment may influence persistence. They do not establish that human vertebral marrow differs from other marrow sites in its dormancy behavior or that the same mechanism operates across breast, prostate, and renal cancer. Figure 1 summarizes this biological sequence while marking the transition to clinical detection as uncertain. Clinical documentation of delayed relapse establishes the timing of recognized disease, whereas experimental niche studies address possible mechanisms of persistence. Neither alone demonstrates continuous dormancy at the vertebral site subsequently involved.

### 5.3. Remodeling and Tumor-Specific Constraints

The marrow environment is dynamic. Experimental work in myeloma showed that osteoclast-mediated remodeling could reactivate dormant cells by altering the endosteal niche [27]. This offers a mechanistic precedent for interactions between remodeling and tumor growth, but myeloma is a marrow malignancy rather than a model of late first recurrence from a solid primary cancer. It should not be used as direct proof that osteoporosis, ordinary exercise, microinjury, or a specific clinical event awakens breast, prostate, or RCC metastases in the spine. Such hypotheses require tumor-specific and site-specific testing.

For prostate cancer, stem-cell niche competition supports skeletal persistence, while clear-cell RCC emphasizes angiogenic and hypoxia-signaling biology [8,9]. The commonality is dependence on a permissive environment; the mechanistic details and clinical evidence are not interchangeable. Targeting a dormancy pathway also poses a translational problem: maintaining quiescence, eliminating persistent cells, and triggering proliferation to increase treatment sensitivity are different objectives with different potential harms. None currently supplies a validated basis for imaging asymptomatic survivors. Biological research is most useful here when it helps formulate measurable questions about recurrence rather than when it is treated as a surrogate for demonstrated surveillance benefit.

## 6. Detection and Surveillance in Long-Term Survivorship

### 6.1. Three Different Purposes of Imaging

Imaging may be used to diagnose symptoms, stage evidence of systemic recurrence, or screen an asymptomatic person without a recurrence signal. These purposes require different evidence. New progressive pain with a cancer history is a diagnostic problem, whereas rising PSA may justify tumor-directed staging. A dedicated spine MRI at a fixed interval in a disease-free survivor is a screening intervention. The fact that MRI can reveal marrow or epidural abnormalities establishes detection capability, not that screening improves function, survival, quality of life, or treatment burden.

MRI, CT, and PET/CT answer complementary questions. MRI depicts marrow replacement, epidural and paraspinal extension, and the relationship of tumor to the cord and cauda equina. CT defines cortical destruction, sclerosis, fracture, and alignment for mechanical assessment and procedural planning. PET/CT provides a whole-body survey of tracer-avid disease, potentially identifying additional skeletal, nodal, or visceral sites. A low-dose CT acquired for PET attenuation correction and localization may not provide the same structural detail as a dedicated diagnostic CT. PET uptake alone does not grade epidural compression or establish stability [28,29].

### 6.2. MRI and PET CT as Complementary Tests

For a survivor with a new suspicious vertebral lesion, MRI addresses local neural risk while tumor-appropriate PET/CT or other staging imaging evaluates disease distribution. Detection of additional sites can change an apparently solitary recurrence into multifocal disease and alter treatment intent. Conversely, a PET-positive vertebral focus may require MRI to establish epidural extent and CT to assess structural compromise. Equivocal findings should be compared with prior examinations and, when management depends on confirmation, evaluated with targeted imaging or biopsy. Suspected cord compression requires urgent MRI and specialist assessment; PET/CT staging must not delay this pathway [28,29].

### 6.3. Molecular Imaging Targets and Tumor Context

2-[18F]FDG-PET/CT reflects glucose utilization and can identify metabolically active skeletal and extraskeletal breast cancer deposits, including marrow lesions without conspicuous structural change. The joint EANM-SNMMI breast guideline supports its use when relapse is clinically suspected, or conventional findings are equivocal, but its principal recommendations concern no special type of breast cancer. Low-grade or lobular tumors and some sclerotic metastases can be less FDG-avid; inflammatory or healing processes can cause false-positive uptake. The CT component therefore remains important even when PET uptake is low [30].

In a prospective comparison involving 18 patients with recurrent breast cancer, Hansen and colleagues found high lesion-based sensitivity of FDG-PET/CT relative to bone scintigraphy or low-dose CT alone; osteolytic lesions were particularly liable to under-detection by scintigraphy. The small patient sample, multiple lesions per patient, and recurrence-enriched population constrain generalization. This evidence supports diagnostic performance in suspected recurrence, not screening effectiveness or a vertebral-specific surveillance interval [31].

[18F]NaF-PET/CT depicts skeletal perfusion and mineralization associated with bone remodeling rather than a tumor-specific target. Its high bone-to-background contrast and PET resolution can reveal remodeling at lesions less conspicuous on planar scintigraphy, including osteoblastic disease. Uptake also occurs in degeneration, fractures, and other benign processes, making CT correlation essential. It cannot directly characterize extraskeletal tumor burden; disease with little reactive bone formation may still be underestimated. In breast or prostate cancer, it may be useful for selected skeletal questions when available, but it is not a universal replacement for tumor-directed PET/CT, MRI, or established staging pathways [32].

Conventional technetium-99m diphosphonate bone scintigraphy likewise reflects blood flow and reactive bone formation. It remains widely available for skeletal assessment in appropriate breast and prostate cancer settings; SPECT/CT can improve localization and characterization of indeterminate uptake. Predominantly osteoblastic lesions can be conspicuous, whereas marrow-predominant or purely lytic deposits with little osteoblastic response may be missed. Degeneration and fracture reduce specificity, and increased uptake after treatment may represent healing flare rather than progression. These limitations require comparison with anatomic imaging and the treatment timeline [33].

PSMA PET/CT uses ligands targeting prostate-specific membrane antigen and can localize both skeletal and extraskeletal prostate cancer, including recurrence detected after a rise in PSA. Performance depends on disease burden, PSA context, tumor expression, and the tracer used. Small or PSMA-low deposits may be occult; benign skeletal uptake, including fracture-related findings, can mimic metastasis. An isolated focus should therefore be interpreted with its CT appearance and, where needed, MRI or follow-up. PSMA PET/CT, NaF-PET/CT, and bone scintigraphy interrogate different biology; their roles depend on the clinical question, availability, and established treatment pathway rather than a single hierarchy of sensitivity [18,34].

Tumor phenotype modifies these comparisons. Breast metastases may be lytic, sclerotic, or mixed; lower FDG uptake does not exclude viable sclerotic disease. Prostate metastases are commonly osteoblastic, favoring visibility with bone-seeking tracers, but PSMA assessment depends on target expression rather than sclerosis alone. RCC metastases are often predominantly lytic, reducing the sensitivity of osteoblast-dependent scintigraphy. Negative bone scintigraphy consequently cannot exclude a clinically suspicious RCC vertebral lesion. MRI and CT remain central to marrow, neural, and structural evaluation, while PET may help resolve selected equivocal findings; limited comparative RCC data do not establish routine PET surveillance [29,30,34]. Table 3 summarizes applications and limitations without assigning a universal numerical sensitivity.

Comparative accuracy also depends on whether studies count lesions or patients, the reference standard, prior treatment, and the spectrum of disease. In proPSMA, PSMA PET/CT improved staging accuracy compared with CT plus bone scintigraphy in newly diagnosed high-risk prostate cancer. That staging trial did not evaluate routine imaging of disease-free survivors or prevention of spinal complications [35]. Improved detection can change recorded stage without proving that the resulting management change improves survival or neurologic outcomes.

### 6.4. Randomized Evidence and Its Applicability

PROMPTS is important because it tested a spinal MRI screening strategy rather than merely documenting the presence of occult disease. The trial randomized 420 patients with metastatic castration-resistant prostate cancer and known asymptomatic spinal metastases. Radiological compression was detected in a substantial proportion of assessable screening scans, but the time-to-clinical-compression comparison did not reach statistical significance; the hazard ratio was 0.64 with a 95% confidence interval of 0.37–1.11 [20]. The confidence interval is compatible with benefit but also with absence of a meaningful reduction. The result should not be described as proof that screening can never help, nor as evidence sufficient to recommend it.

PROMPTS enrolled patients with established metastatic castration-resistant prostate cancer, not disease-free survivors after curative-intent treatment. Its intervention sought to identify occult radiological compression in patients with known spinal metastases; it did not seek to detect the first vertebral recurrence in survivors without diagnosed distant disease. Baseline lesion prevalence, systemic treatment exposure, competing mortality, and opportunities for intervention therefore differ substantially between these populations. Its effect estimate and screening yield cannot be transferred directly to disease-free survivors. Nevertheless, the trial shows why identifying radiological abnormalities does not by itself establish a clinically beneficial screening program. NICE advises against MRI solely for early radiological detection of cord compression in patients with known spinal metastases who lack symptoms or signs of compression [28]. The absence of a statistically significant benefit in PROMPTS neither proves that screening is ineffective in every population nor establishes a rationale for screening disease-free survivors. Direct evidence in the latter population remains insufficient.

Gillespie and colleagues randomized patients with established metastatic solid tumors and selected high-risk asymptomatic bone lesions to prophylactic radiation or standard care and observed fewer skeletal-related events with radiation [21]. This is evidence with regard to treating an already identified high-risk lesion. It does not test how to find such lesions among disease-free survivors, does not establish a spine-screening interval, and does not mean that all asymptomatic vertebral metastases require radiation. Table 2 places this trial alongside PROMPTS and ORIOLE because their different entry populations and interventions answer different questions.

### 6.5. Benefits, Harms, and Bias

Earlier identification could be valuable if it reveals a lesion before irreversible neural injury or mechanical failure, when an effective and tolerable intervention is available. That possibility is strongest as a research hypothesis, because screening benefit depends on more than lesion detectability. The event must occur often enough in the selected population, the imaging window must precede harm, and treatment prompted by screening must improve outcomes compared with treatment prompted by ordinary clinical evaluation. A sensitive test may increase recorded prevalence without satisfying these conditions.

An asymptomatic metastasis is not automatically an actionable vertebral event. Actionability requires sufficient diagnostic confidence and a management consequence expected to benefit the patient: evidence of systemic recurrence may change systemic therapy, additional sites may alter eligibility for metastasis-directed treatment, and local epidural extension or instability may prompt radiation, decompression, or stabilization. PET/CT can map the extent of recurrence and help select a biopsy target, while MRI and CT establish local threats. A small stable focus without neural compromise may instead prompt confirmation, systemic management, or observation. ORIOLE and the trial of prophylactic radiation to high-risk bone lesions illustrate treatment opportunities after selected disease has been identified; they do not establish a benefit from searching for such lesions in all survivors [19,21]. A higher detection rate or a changed treatment plan is an intermediate endpoint, not evidence by itself of longer survival, fewer fractures, or fewer neurologic complications.

Lead-time bias can make survival after detection appear longer without delaying death or disability. Length bias can preferentially identify indolent disease, creating an impression that screened patients do better because of screening. Incidental abnormalities can trigger repeat imaging, biopsy, or treatment without a corresponding benefit. These considerations are especially relevant in older survivors with degenerative changes and fragility fractures. Risk-adapted imaging should therefore be evaluated against patient-centered outcomes and downstream consequences rather than justified solely by increased lesion detection or earlier radiological staging.

## 7. Clinical Evaluation of a Suspected or Newly Detected Lesion

### 7.1. Symptoms and Diagnostic Urgency

Remote cancer history should remain part of the assessment when a survivor develops new, progressive, nocturnal, or mechanical spinal pain. Persistent severe pain, pain that disturbs sleep, or pain aggravated by movement warrants prompt clinical assessment. NICE recommends seeking advice through the metastatic spinal cord compression (MSCC) coordinator within 24 h for a person with past or current cancer and pain suggestive of spinal metastases. When spinal metastases are clinically suspected without suspected cord compression, MRI should be obtained within one week [28]. Local oncology and spine referral pathways should provide equivalent timely access.

New weakness, gait disturbance, sensory loss, or bladder or bowel dysfunction raises concern for neural compression and requires immediate contact with the MSCC service or the corresponding emergency oncology and spine team. Suspected MSCC is an oncological emergency: MRI should be performed as soon as possible and always within 24 h, without delaying referral for completion of routine outpatient staging [28]. Neurologic deterioration requires immediate escalation; the 24 h benchmark is not a reason to defer an available urgent assessment. Emergency diagnostic evaluation must remain distinct from elective imaging of asymptomatic disease-free survivors.

The initial evaluation should define symptom duration, progression, pain with loading, neurologic function, prior radiation or spinal procedures, and the original cancer history. Imaging and referral should proceed together when delay could affect function. For an incidental lesion without red flags, the task is to clarify diagnosis and extent, compare prior imaging, and determine whether systemic recurrence is present. An atypical solitary lesion after a prolonged interval may warrant biopsy before a treatment plan is finalized. The choice depends on diagnostic uncertainty, procedural feasibility, and whether the result will change management.

### 7.2. Neurologic and Mechanical Assessment

After detection, NOMS provides a useful framework for integrating neurologic, oncologic, mechanical, and systemic factors [5]. The neurologic assessment combines clinical function with epidural extent rather than using either alone. Imaging may show epidural disease in a patient without deficits, while symptoms can demand urgent assessment before complete staging is available. The extent of compression, tumor sensitivity to radiation, and feasibility of rapid treatment influence whether decompression is needed. A long disease-free interval does not protect against the consequences of a lesion that has already reached the epidural space.

The epidural spinal cord compression scale offers a standardized MRI description of epidural extent. Bilsky and colleagues demonstrated good to excellent interobserver and intraobserver reliability when grading axial T2-weighted images [36]. Used alongside the neurologic examination, the scale helps communicate the relationship between the tumor, cerebrospinal fluid, and cord. It describes local disease severity rather than systemic prognosis or mechanical stability, and should be integrated with the remaining NOMS domains rather than used alone to select treatment.

Mechanical instability is independently important. SINS incorporates location, mechanical pain, lesion quality, alignment, collapse, and posterolateral involvement to support a shared assessment across specialties [37]. It is a communication and referral tool, not a stand-alone instruction for a particular operation. Radiation can control the tumor but does not immediately reconstruct a failed load-bearing segment. Conversely, a stable vertebra without neural compromise may be managed without surgery. Distinguishing pain caused by instability from other pain mechanisms helps avoid assuming that all symptoms will respond to the same local treatment.

### 7.3. Local Treatment in the Context of Systemic Disease

The oncologic domain considers histology, prior therapy, radiosensitivity, local disease-control goals, and systemic options. The systemic domain considers disease distribution, medical fitness, expected survival, and patient priorities [5]. Detection of an apparently solitary lesion should prompt appropriate staging because additional disease may alter treatment intent. Even with limited burden, preservation of ambulation and avoidance of prolonged recovery may matter more to a patient than maximal local intervention. Shared decision-making should specify the functional objective and the expected burden of the proposed treatment.

Randomized evidence supports some local interventions in selected populations. Patchell and colleagues found better functional outcomes with decompressive surgery followed by radiation than with radiation alone in selected patients with metastatic cord compression [22]. SC.24 showed a higher complete pain-response rate with a specified stereotactic regimen than with conventional radiation in selected patients with painful spinal metastases [23]. These results support appropriately selected treatment, not a universal preference for surgery or stereotactic treatment after late recurrence. Eligibility, compression, stability, previous irradiation, and goals must be considered. Neither trial demonstrates that routine imaging of asymptomatic long-term survivors will create the same benefit.

The radiation evidence also requires attention to regimen and endpoint. In NRG Oncology/RTOG 0631, single-fraction stereotactic radiosurgery at 16 or 18 Gy did not improve the primary three-month pain-response outcome compared with conventional 8-Gy radiation [24]. This result differs from the complete pain-response benefit reported with 24 Gy in two fractions in SC.24; the trials used different treatment schedules, eligibility criteria, and response definitions. Their findings should not be collapsed into a general claim that every stereotactic regimen is superior. The 2024 ASTRO guideline addresses conventional radiation, selected use of stereotactic treatment, and reirradiation for symptomatic bone metastases, emphasizing patient and disease factors in treatment selection [38]. This guidance supports individualized management of detected disease and does not establish routine screening of disease-free survivors.

## 8. Survivorship Coordination and Research Priorities

### 8.1. Coordination Across the Care Pathway

**Figure 2:**
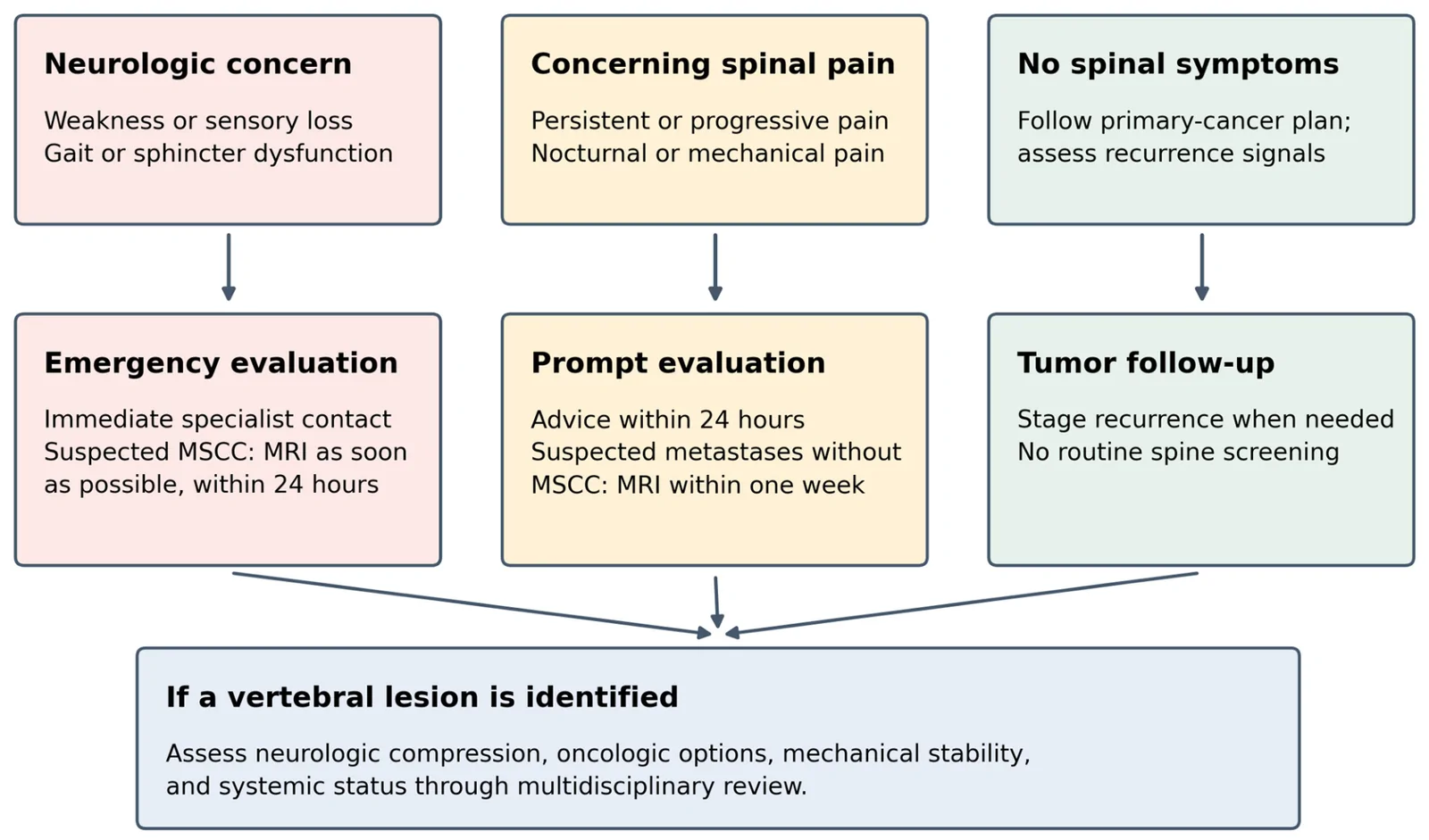
**Clinical evaluation pathways.** Conceptual pathway for survivors of solid cancer, adapted from NICE NG234 [28]. Neurologic symptoms or signs suggesting metastatic spinal cord compression require immediate specialist contact and MRI as soon as possible, always within 24 h. Pain suggesting spinal metastases without suspected compression warrants specialist advice within 24 h and MRI within one week. These diagnostic pathways are distinct from scheduled screening of asymptomatic survivors. A detected lesion requires multidisciplinary assessment. This is not a validated risk algorithm.

Long-term survivors may move between oncology, primary care, and specialty clinics, making it important that the original diagnosis and treatment remain accessible when new symptoms occur. A survivorship plan should identify who evaluates persistent spinal symptoms, how urgent neurologic concerns are escalated, and how new imaging findings reach the treating oncology team. This is a practical coordination proposal rather than a new screening standard. The clinical pathway in Figure 2 distinguishes symptom-triggered investigation, tumor-specific recurrence staging, and routine follow-up so that each has an explicit purpose.

Patient education should encourage reporting of progressive pain and neurologic changes without suggesting that ordinary back pain necessarily signals cancer. After treatment of a vertebral lesion, rehabilitation, pain control, bone health, and follow-up remain important to preserving function. Communication should also clarify responsibility for monitoring local disease and systemic treatment response. A survivorship-oriented perspective therefore extends beyond detecting a lesion: it includes reducing delays in appropriate assessment and supporting recovery after recurrence while avoiding unnecessary investigation of stable, low-concern findings.

### 8.2. Building Vertebral-Specific Evidence

A prospective registry should enroll patients at a defined disease-free landmark and retain primary stage, histology, molecular features, treatment exposure, and time since treatment. Each recurrence should record whether the vertebra was involved at first distant relapse, whether other sites were simultaneous, and how the lesion was discovered. Presentation should include pain, neurologic function, fracture or instability, epidural involvement, and urgency of intervention. Recording only the diagnosis of bone metastasis cannot connect detection with the morbidity that spine-aware care seeks to prevent.

Competing mortality, loss to follow-up, changes in imaging technology, and treatment-era effects must be considered when estimating conditional risk. A model predicting any distant recurrence may not predict an actionable vertebral event. External validation is needed across institutions and survivorship populations, followed by assessment of whether the model changes care beneficially. Molecular residual-disease markers and other candidate biomarkers may eventually help enrich a population, but localization and clinical actionability still require evaluation. Their potential should be treated as a research direction rather than a current indication for serial spinal imaging.

Molecular imaging could be evaluated within prospective risk-adapted surveillance studies, with eligibility defined by tumor biology, recurrence markers, and time since treatment. Protocols should specify the tracer, scan interval, interpretation thresholds, and management of equivocal uptake, and distinguish PSA-triggered recurrence staging from imaging without a recurrence signal. MRI and CT confirmation should document vertebral location, epidural extent, and structural consequences. Prespecified outcomes should include actionable findings, false-positive investigations and biopsies, cumulative radiation exposure, anxiety, access, cost-effectiveness, and subsequent neurologic or skeletal events. Increased lesion yield would justify further study, but adoption would require a favorable balance of patient benefit, harms, and resource use.

Interventional studies should measure neurologic function, unplanned decompression or stabilization, fracture, quality of life, treatment burden, false-positive investigations, and cost. A trial should also distinguish the effect of imaging from the effect of treatment after detection, document adherence to both strategies, and account for competing events. The essential question is whether a defined imaging-and-treatment pathway prevents meaningful harm relative to timely clinical evaluation. Merely shortening the interval to diagnosis or increasing the number of lesions treated would not establish that benefit.

## 9. Limitations

This narrative synthesis is selective, limited to the assessed English-language literature, and does not establish a comprehensive evidence inventory or a pooled incidence estimate. Qualitative study selection and the absence of independent duplicate screening create a risk of selection bias. Imaging accuracy studies also differ in patient spectrum, reference standards, and lesion-based versus patient-based analyses. Historical long-term cohorts, modern molecular-imaging studies, and advanced-disease trials describe different populations. Experimental niche studies support biological hypotheses but cannot determine the timing or origin of an individual human vertebral lesion. Guideline recommendations also address specific disease states and should not be extrapolated without considering their scope. These limitations favor a cautious interpretation of surveillance proposals while supporting prompt diagnostic assessment when symptoms or evidence of recurrence arise.

## 10. Conclusions

Late vertebral metastasis is a consequential possibility after breast, prostate, or renal cell cancer, but the risk of a first late vertebral event is not adequately captured by existing bone-metastasis reporting. Tumor-specific recurrence trajectories and follow-up practices must be distinguished, as must first relapse, biochemical recurrence, and progression of established metastatic disease. Marrow-persistence mechanisms provide biological plausibility without proving a uniquely vertebral dormancy process. Current evidence does not establish a role for scheduled spine MRI in asymptomatic disease-free survivors. Improved PET/CT detection likewise does not establish a benefit from routine molecular imaging surveillance. Concerning symptoms and tumor-specific recurrence signals warrant timely evaluation; identified lesions require coordinated assessment of neural compression, stability, treatment options, and systemic status. Prospective vertebral-specific cohorts and appropriately designed intervention trials are needed to determine whether risk-defined detection can prevent morbidity while limiting unnecessary investigation and treatment.

## Figures and Tables

**Table 1:** Late recurrence and vertebral considerations.

Feature	HR-Positive Breast Cancer	Prostate Adenocarcinoma	Renal Cell Carcinoma
Late-recurrence evidence	Persistent distant risk in years 5–20; risk varies by initial stage [1].	Late biochemical or metastatic recurrence may follow local treatment [4].	Recurrence can occur beyond 5 or 10 years; cohort denominator matters [2,3].
Vertebral endpoint	Often aggregated with other bone sites.	Lesions may be identified during recurrence staging.	Bone events often reported without vertebral detail.
Typical lesion pattern	Lytic, mixed, or sclerotic.	Often sclerotic; appearance alone does not establish stability.	Often lytic; potential vascularity affects procedural planning.
Mechanistic evidence	Experimental marrow and vascular niche studies [6,7].	Mouse stem-cell niche competition [8].	Established VHL/HIF biology; direct vertebral dormancy evidence limited [9].
Follow-up context	Clinical and breast follow-up; no routine systemic imaging in asymptomatic patients [10].	PSA follow-up; image recurrence when the result will affect care [4].	Risk-based oncologic imaging and individualized duration [11].
Clinical implication	Investigate persistent concerning spinal symptoms.	Separate biochemical staging from spine-specific assessment.	Retain remote RCC history in the differential of a new lesion.

HR = hormone receptor; PSA = prostate-specific antigen; VHL = von Hippel–Lindau; HIF = hypoxia-inducible factor. Entries distinguish systemic recurrence evidence from vertebral-specific evidence and do not constitute screening recommendations.

**Table 2:** Key evidence and its clinical applicability.

Evidence	Population and Question	Contribution	Limit of Applicability
Pan et al. [1]	ER-positive breast cancer after planned 5-year endocrine treatment; late distant recurrence.	Demonstrates sustained systemic recurrence risk.	Does not estimate first late vertebral recurrence or test imaging.
Kim and Miyao et al. [2,3]	RCC after nephrectomy; late events in long-term cohorts.	Documents recurrence after prolonged intervals.	Different landmarks and denominators; bone is not a vertebral endpoint.
PROMPTS [20]	Known asymptomatic spinal metastases in castration-resistant prostate cancer; screening MRI.	No statistically significant reduction in clinical compression.	Advanced-disease population differs from disease-free survivors.
Gillespie et al. [21]	Established metastatic cancer with high-risk asymptomatic bone lesions; prophylactic radiation.	Supports selected intervention after lesion identification.	Does not test a survivor-screening strategy.
ORIOLE [19]	Recurrent hormone-sensitive oligometastatic prostate cancer; ablative radiation.	Informs treatment of selected detected metastases.	Not a spine-specific screening or compression-prevention trial.
Patchell, SC.24, and RTOG 0631 [22,23,24]	Selected patients with compression or painful spinal metastases; local treatment.	Informs selected local treatment; radiation outcomes vary by regimen and population.	Treatment efficacy does not establish a benefit from screening.

ER = estrogen receptor. Studies answer distinct questions about recurrence, screening, and treatment; their findings should not be transferred across populations without qualification.

**Table 3:** Imaging modalities for suspected vertebral metastases.

Modality and Target	Principal Application	Advantages	Important Limitations
MRI Marrow and soft tissue	Marrow disease; epidural extent; suspected neural compression [28,29].	Defines cord, cauda equina, and local soft tissue relationships; no ionizing radiation.	Availability and patient/device constraints; targeted spine MRI is not whole-body staging.
CT Mineralized bone	Destruction, sclerosis, collapse, alignment; procedural planning [29].	Detailed cortical and structural assessment.	Early marrow disease may be inconspicuous; limited neural detail; ionizing radiation.
Bone scintigraphy 99mTc diphosphonate	Skeletal survey in appropriate cancer staging or recurrence settings [33].	Widely available; depicts reactive bone formation; SPECT/CT improves localization.	Benign uptake and healing flare; limited detection of purely lytic or marrow-only disease.
2-[18F]FDG-PET/CT Glucose utilization	Whole-body evaluation of suspected breast cancer recurrence [30,31].	Detects active skeletal and extraskeletal disease; may reveal CT-occult marrow lesions.	Variable uptake in low-grade, lobular, or sclerotic disease; inflammation; radiation.
[18F]NaF-PET/CT Bone remodeling	Selected assessment of skeletal involvement [32].	High contrast and spatial resolution for active skeletal remodeling.	Not tumor-specific; benign uptake; no direct extraskeletal tumor assessment; cost and access.
PSMA PET/CT PSMA expression	Prostate cancer staging and biochemical recurrence evaluation [34,35].	Maps target-expressing bone, nodal, and visceral disease.	PSMA-low/small lesions can be missed; benign uptake and tracer-specific pitfalls; radiation.

FDG = fluorodeoxyglucose; NaF = sodium fluoride; PSMA = prostate-specific membrane antigen; SPECT = single-photon emission computed tomography. Modalities are complementary. None of the diagnostic performance evidence summarized here establishes routine imaging surveillance in asymptomatic disease-free survivors.

## Data Availability

No new data were created or analyzed in this study. Data sharing is not applicable to this article.

## References

[1] Pan H., Gray R., Braybrooke J., Davies C., Taylor C., McGale P., Peto R., Pritchard K.I., Bergh J., Dowsett M. (2017). 20-Year Risks of Breast-Cancer Recurrence after Stopping Endocrine Therapy at 5 Years. N. Engl. J. Med..

[2] Kim S.P., Weight C.J., Leibovich B.C., Thompson R.H., Costello B.A., Cheville J.C., Lohse C.M., Boorjian S.A. (2011). Outcomes and clinicopathologic variables associated with late recurrence after nephrectomy for localized renal cell carcinoma. Urology.

[3] Miyao N., Naito S., Ozono S., Shinohara N., Masumori N., Igarashi T., Nakao M., Tsushima T., Senga Y., Horie S. (2011). Late recurrence of renal cell carcinoma: Retrospective and collaborative study of the Japanese Society of Renal Cancer. Urology.

[4] European Association of Urology (2026). EAU Guidelines on Prostate Cancer: Follow-Up. https://uroweb.org/guidelines/prostate-cancer/chapter/followup.

[5] Laufer I., Rubin D.G., Lis E., Cox B.W., Stubblefield M.D., Yamada Y., Bilsky M.H. (2013). The NOMS framework: Approach to the treatment of spinal metastatic tumors. Oncologist.

[6] Price T.T., Burness M.L., Sivan A., Warner M.J., Cheng R., Lee C.H., Olivere L., Comatas K., Magnani J., Lyerly H.K. (2016). Dormant breast cancer micrometastases reside in specific bone marrow niches that regulate their transit to and from bone. Sci. Transl. Med..

[7] Ghajar C.M., Peinado H., Mori H., Matei I.R., Evason K.J., Brazier H., Almeida D., Koller A., Hajjar K.A., Stainier D.Y. (2013). The perivascular niche regulates breast tumour dormancy. Nat. Cell Biol..

[8] Shiozawa Y., Pedersen E.A., Havens A.M., Jung Y., Mishra A., Joseph J., Kim J.K., Patel L.R., Ying C., Ziegler A.M. (2011). Human prostate cancer metastases target the hematopoietic stem cell niche to establish footholds in mouse bone marrow. J. Clin. Investig..

[9] Shirole N.H., Kaelin W.G. (2023). von-Hippel Lindau and Hypoxia-Inducible Factor at the Center of Renal Cell Carcinoma Biology. Hematol. Oncol. Clin. N. Am..

[10] Khatcheressian J.L., Hurley P., Bantug E., Esserman L.J., Grunfeld E., Halberg F., Hantel A., Henry N.L., Muss H.B., Smith T.J. (2013). Breast cancer follow-up and management after primary treatment: American Society of Clinical Oncology clinical practice guideline update. J. Clin. Oncol..

[11] European Association of Urology (2026). EAU Guidelines on Renal Cell Carcinoma: Follow-Up in RCC. https://uroweb.org/guidelines/renal-cell-carcinoma/chapter/followup-in-rcc.

[12] Pedersen R.N., Esen B.Ö., Mellemkjær L., Christiansen P., Ejlertsen B., Lash T.L., Nørgaard M., Cronin-Fenton D. (2022). The Incidence of Breast Cancer Recurrence 10–32 Years After Primary Diagnosis. J. Natl. Cancer Inst..

[13] Rueda O.M., Sammut S.-J., Seoane J.A., Chin S.-F., Caswell-Jin J.L., Callari M., Batra R., Pereira B., Bruna A., Ali H.R. (2019). Dynamics of breast-cancer relapse reveal late-recurring ER-positive genomic subgroups. Nature.

[14] Davies C., Pan H., Godwin J., Gray R., Arriagada R., Raina V., Abraham M., Medeiros Alencar V.H., Badran A., Bonfill X. (2013). Long-term effects of continuing adjuvant tamoxifen to 10 years versus stopping at 5 years after diagnosis of oestrogen receptor-positive breast cancer: ATLAS, a randomised trial. Lancet.

[15] Goss P.E., Ingle J.N., Pritchard K.I., Robert N.J., Muss H., Gralow J., Gelmon K., Whelan T., Strasser-Weippl K., Rubin S. (2016). Extending Aromatase-Inhibitor Adjuvant Therapy to 10 Years. N. Engl. J. Med..

[16] Braun S., Vogl F.D., Naume B., Janni W., Osborne M.P., Coombes R.C., Schlimok G., Diel I.J., Gerber B., Gebauer G. (2005). A pooled analysis of bone marrow micrometastasis in breast cancer. N. Engl. J. Med..

[17] (2015). Early Breast Cancer Trialists’ Collaborative Group. Adjuvant bisphosphonate treatment in early breast cancer: Meta-analyses of individual patient data from randomised trials. Lancet.

[18] Fendler W.P., Calais J., Eiber M., Flavell R.R., Mishoe A., Feng F.Y., Nguyen H.G., Reiter R.E., Rettig M.B., Okamoto S. (2019). Assessment of 68Ga-PSMA-11 PET Accuracy in Localizing Recurrent Prostate Cancer: A Prospective Single-Arm Clinical Trial. JAMA Oncol..

[19] Phillips R., Shi W.Y., Deek M., Radwan N., Lim S.J., Antonarakis E.S., Rowe S.P., Ross A.E., Gorin M.A., Deville C. (2020). Outcomes of Observation vs Stereotactic Ablative Radiation for Oligometastatic Prostate Cancer: The ORIOLE Phase 2 Randomized Clinical Trial. JAMA Oncol..

[20] Dearnaley D., Hinder V., Hijab A., Horan G., Srihari N., Rich P., Houston J.G., Henry A.M., Gibbs S., Venkitaraman R. (2022). Observation versus screening spinal MRI and pre-emptive treatment for spinal cord compression in patients with castration-resistant prostate cancer and spinal metastases in the UK (PROMPTS): An open-label, randomised, controlled, phase 3 trial. Lancet Oncol..

[21] Gillespie E.F., Yang J.C., Mathis N.J., Marine C.B., White C., Zhang Z., Barker C.A., Kotecha R., McIntosh A., Vaynrub M. (2024). Prophylactic Radiation Therapy Versus Standard of Care for Patients with High-Risk Asymptomatic Bone Metastases: A Multicenter, Randomized Phase II Clinical Trial. J. Clin. Oncol..

[22] Patchell R.A., Tibbs P.A., Regine W.F., Payne R., Saris S., Kryscio R.J., Mohiuddin M., Young B. (2005). Direct decompressive surgical resection in the treatment of spinal cord compression caused by metastatic cancer: A randomised trial. Lancet.

[23] Sahgal A., Myrehaug S.D., Siva S., Masucci G.L., Maralani P.J., Brundage M., Butler J., Chow E., Fehlings M.G., Foote M. (2021). Stereotactic body radiotherapy versus conventional external beam radiotherapy in patients with painful spinal metastases: An open-label, multicentre, randomised, controlled, phase 2/3 trial. Lancet Oncol..

[24] Ryu S., Deshmukh S., Timmerman R.D., Movsas B., Gerszten P., Yin F.-F., Dicker A., Abraham C.D., Zhong J., Shiao S.L. (2023). Stereotactic Radiosurgery vs. Conventional Radiotherapy for Localized Vertebral Metastases of the Spine: Phase 3 Results of NRG Oncology/RTOG 0631 Randomized Clinical Trial. JAMA Oncol..

[25] Kim J., Ham W.S., Park J.S., Jang W.S. (2024). Incidence and Pattern of Recurrence after Surgical Resection in Organ-Confined Renal Cell Carcinoma. Yonsei Med. J..

[26] Batson O.V. (1940). The function of the vertebral veins and their role in the spread of metastases. Ann. Surg..

[27] Lawson M.A., McDonald M.M., Kovacic N., Khoo W.H., Terry R.L., Down J., Kaplan W., Paton-Hough J., Fellows C., Pettitt J.A. (2015). Osteoclasts control reactivation of dormant myeloma cells by remodelling the endosteal niche. Nat. Commun..

[28] National Institute for Health and Care Excellence (2023). Spinal Metastases and Metastatic Spinal Cord Compression. NG234. https://www.nice.org.uk/guidance/ng234.

[29] Grünwald V., Eberhardt B., Bex A., Flörcken A., Gauler T., Derlin T., Panzica M., Dürr H.R., Grötz K.A., Giles R.H. (2018). An interdisciplinary consensus on the management of bone metastases from renal cell carcinoma. Nat. Rev. Urol..

[30] Vaz S.C., Woll J.P.P., Cardoso F., Groheux D., Cook G.J.R., Ulaner G.A., Jacene H., Rubio I.T., Schoones J.W., Peeters M.-J.V. (2024). Joint EANM-SNMMI guideline on the role of 2-[18F]FDG PET/CT in no special type breast cancer: (endorsed by the ACR, ESSO, ESTRO, EUSOBI/ESR, and EUSOMA). Eur. J. Nucl. Med. Mol. Imaging.

[31] Hansen J.A., Naghavi-Behzad M., Gerke O., Baun C., Falch K., Duvnjak S., Alavi A., Høilund-Carlsen P.F., Hildebrandt M.G. (2021). Diagnosis of bone metastases in breast cancer: Lesion-based sensitivity of dual-time-point FDG-PET/CT compared to low-dose CT and bone scintigraphy. PLoS ONE.

[32] Beheshti M., Mottaghy F.M., Payche F., Behrendt F.F.F., Wyngaert T.V.D., Fogelman I., Strobel K., Celli M., Fanti S., Giammarile F. (2015). 18F-NaF PET/CT: EANM procedure guidelines for bone imaging. Eur. J. Nucl. Med. Mol. Imaging.

[33] Van den Wyngaert T., Strobel K., Kampen W.U., Kuwert T., Van Der Bruggen W., Mohan H.K., Gnanasegaran G., Delgado-Bolton R., Weber W.A., Beheshti M. (2016). The EANM practice guidelines for bone scintigraphy. Eur. J. Nucl. Med. Mol. Imaging.

[34] Fendler W.P., Eiber M., Beheshti M., Bomanji J., Calais J., Ceci F., Cho S.Y., Fanti S., Giesel F.L., Goffin K. (2023). PSMA PET/CT: Joint EANM procedure guideline/SNMMI procedure standard for prostate cancer imaging 2.0. Eur. J. Nucl. Med. Mol. Imaging.

[35] Hofman M.S., Lawrentschuk N., Francis R.J., Tang C., Vela I., Thomas P., Rutherford N., Martin J.M., Frydenberg M., Shakher R. (2020). Prostate-specific membrane antigen PET-CT in patients with high-risk prostate cancer before curative-intent surgery or radiotherapy (proPSMA): A prospective, randomised, multicentre study. Lancet.

[36] Bilsky M.H., Laufer I., Fourney D.R., Groff M., Schmidt M.H., Varga P.P., Vrionis F.D., Yamada Y., Gerszten P.C., Kuklo T.R. (2010). Reliability analysis of the epidural spinal cord compression scale. J. Neurosurg. Spine.

[37] Fourney D.R., Frangou E.M., Ryken T.C., DiPaola C.P., Shaffrey C.I., Berven S.H., Bilsky M.H., Harrop J.S., Fehlings M.G., Boriani S. (2011). Spinal instability neoplastic score: An analysis of reliability and validity from the Spine Oncology Study Group. J. Clin. Oncol..

[38] Alcorn S., Cortés Á.A., Bradfield L., Brennan M., Dennis K., Diaz D.A., Doung Y.-C., Elmore S., Hertan L., Johnstone C. (2024). External Beam Radiation Therapy for Palliation of Symptomatic Bone Metastases: An ASTRO Clinical Practice Guideline. Pract. Radiat. Oncol..

